# A scaffolded training series to develop Clinical and Translational Researchers in Hawai‘i

**DOI:** 10.1017/cts.2025.10108

**Published:** 2025-08-01

**Authors:** Merle Kataoka-Yahiro, F. David Horgen, Dedra Buchwald, Claire Townsend Ing, Spero M. Manson, Joseph Keawe‘aimoku Kaholokula, Kathryn L. Braun

**Affiliations:** 1 Department of Nursing, School of Nursing and Dental Hygiene, University of Hawai‘i at Mānoa, Honolulu, HI, USA; 2 Chemistry and Biochemistry Program, Hawai‘i Pacific University, Honolulu, HI, USA; 3 UW Medicine and Neurosciences Institute, University of Washington, Seattle, WA, USA; 4 Centers for American Indian and Alaska Native Health, University of Colorado, Aurora, CO, USA; 5 Department of Native Hawaiian Health, John A. Burns School of Medicine, University of Hawai‘i at Mānoa, Honolulu, HI, USA; 6 Office of Public Health Studies, Thompson School of Social Work & Public Health, University of Hawai‘i at Mānoa, Honolulu, HI, USA

**Keywords:** Clinical translational research, mentoring, career development, research education and training, scaffolding

## Abstract

We describe a training series using a scaffolded approach informed by Vygotsky’s Learning Theory to advance Hawai‘i -based faculty grant-writing skills. Sponsored by the Professional Development Core of the Center for Pacific Innovations, Knowledge, and Opportunities (PIKO) at the University of Hawai‘i at Mānoa, the initiative includes a 2-week series of 1-hour introductory sessions on aspects of grant writing, a 3-session workshop to develop a specific aims page, and a 5-month training program in grant writing. Over three years, 202 Hawai‘i investigators attended at least one 1-hour introductory session, 62 completed the workshop on preparing specific aims, and 30 completed the 5-month training on grant writing. Participants rated all 3 programs as very useful. Of the 62 unique investigators who completed the Specific Aims Workshop, 21 (33%) submitted PIKO pilot grant applications, 4 (6%) submitted grants elsewhere, and 16 (30%) applied to the 5-month training on grant writing. The 30 GUMSHOE participants reported significant gains in their confidence in accomplishing 21 proposal-writing tasks and, as of May 2025, 26 (87%) submitted grants to the National Institutes of Health or another external funder. This scaffolded training approach is labor- and time-intensive for trainees and faculty mentors, but our outcomes demonstrate its success.

## Introduction

Hawai‘i is one of 24 eligible states and territories in the US with historically low levels of funding from the National Institutes of Health (NIH). Thus, Hawai‘i is eligible to apply for support under the NIH-funded Institutional Development Award (IDeA), which is a ‘congressionally mandated program that builds research capacity in states with limited NIH funding [1].” The IDeA program “aims to strengthen an institution’s ability to support biomedical research, enhance the competitiveness of investigators in securing research funding, and enable clinical and translational research [1].”

In 2021, the University of Hawai‘i was awarded an IDeA Clinical and Translational Research (CTR) center (U54GM138062), called The Center for Pacific Innovations, Knowledge, and Opportunities (PIKO), to strengthen the statewide CTR infrastructure. PIKO is a partnership across the University of Hawai‘i, Hawai‘i Pacific University, Chaminade University of Honolulu, and a statewide network of 18 practice- and community-based organizations. PIKO is organized around seven cores that provide support to the CTR research community, namely, Professional Development (PD); Pilot Projects (which awards funds to 5+ pilot projects each year); Biostatistics, Epidemiology, and Research Design; Community Engagement and Outreach; Clinical Research and Regulatory Services; Tracking and Evaluation; and Administration.

The PD Core directly supports the growth, effectiveness, and sustainability of the CTR workforce. Hawai‘i-based investigators can seek professional development services from PIKO’s PD Core, including career advice and mentoring, links to potential mentors and partners, development of Individual Development Plans (IDP), assistance in writing manuscripts, and training in specific funding mechanisms and grant writing.

**Figure 1.**
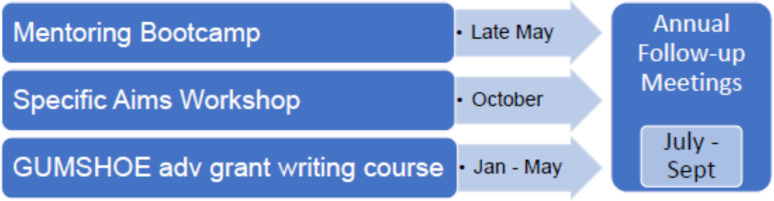
PIKO scaffolded training series. Mentoring Bootcamp, a series of 1-hour sessions that introduce participants to grant writing and research management. Specific Aims Workshop, a multi-session program to help investigators develop a specific aims page. GUMSHOE – Grant-writing Uncovered: Maximizing Strategies, Help, Opportunities, Experiences, a 5-month training in grant writing.

This paper describes the training series that comprise the PIKO PD Core’s unique scaffolded approach to train investigators in grant writing. The series includes: (1) Mentoring Bootcamp, a series of 1-hour sessions that introduce participants to NIH grant writing and research management; (2) the Specific Aims Workshop, a multi-session program to help investigators develop a specific aims page; (3) GUMSHOE (Grant-writing-Uncovered: Maximizing Strategies, Help, Opportunities, Experience), a 5-month training in grant writing, and (4) annual personalized mentoring meetings (Figure 1). The training components were developed based on an assessment of faculty needs and our collective experiences as teachers of grant writing.

### Conceptual framework

The scaffolded approach is rooted in a sociocultural perspective, the main tenets of which derive from Vygotsky’s Learning Theory [2]. Vygotsky viewed learning as taking place between experts and novices in a sociocultural context that increases the latter’s independence while diminishing expert support over time. Novice learners practice skills, receive feedback, and are supported by experts at each step of the grant writing process [3,4]. This method of active learning maximizes non-threatening, highly relevant, incremental success that gradually reduces the role of experts as mentees become independent learners and researchers [5]. Vygotsky’s ideas support the transference and internalization of new knowledge through collaborative learning experiences, reciprocal teaching, and social interaction [3–5].

Macro-, meso-, and micro-scaffolding were incorporated into our training series. Macro-scaffolding refers to the intentional organization of the overall sequence of training and mentoring opportunities to support learning. This involves the progression of content and tasks over multiple courses, moving from broad concepts to specific, hands-on applications. Meso-scaffolding pertains to the thoughtful structuring of content and activities to guide novices’ knowledge acquisition and to refine their skills, starting with simple tasks that gradually build to more complex assignments. Micro-scaffolding refers to the “just-in-time” support provided by experts to help novices master complex tasks, offering immediate assistance as needed to enhance their learning and enable them to eventually undertake skills independently [5].

This approach aligns with the active learning model adopted by the NIH National Research Mentoring Network, which has been shown to build self-efficacy with grant writing [6–8]. It also aligns with models that personalize mentoring in consideration of the mentees’ environment and culture [9–12], including the Native Investigator Development Program, which was tailored for early-stage investigators working in native communities [10,11]. The efforts of the PIKO PD Core described here also build on earlier mentoring programs for Hawai‘i’s investigators [13–15], while expanding opportunities for personalized mentoring, skills, practice, and peer review in NIH grant writing.

Importantly, this learning process operates within a particular community setting. Professional development includes developing the CTR workforce, i.e., interdisciplinary teams comprised of academic and community researchers. Professional development is also about developing a “thinkforce” that will ensure the continuing contribution of the lived experiences and perspectives of individuals from academic, clinical, and community backgrounds to scientific endeavors [16].

### Training components

Our scaffolded approach includes three PIKO PD Core trainings of increasing intensity offered over a year: (1) Mentoring Bootcamp, (2) Specific Aims Workshop, and (3) GUMSHOE. Although it is not required that investigators attend the three training components in a specific order, the series is designed to lead investigators from general knowledge about elements of a grant in Bootcamp to more in-depth learning and practice of grant writing skills in the Specific Aims Workshop and GUMSHOE. At the end of each cycle, the PD Core conducts personalized mentoring meetings with PIKO investigators to discuss their progress toward their professional development goals and grant completion and submission.

**Mentoring Bootcamp** is a program of Ola HAWAII (U54MD007601), the state’s Research Center in Minority Institutions (RCMI). PIKO has co-sponsored Mentoring Bootcamp since 2022. The program is offered in 2-hour blocks, 3 days a week over 2 weeks in May, timed to occur after classes end and before faculty on 9-month appointments disperse for the summer. More than 24 presenters deliver 1-hour sessions that introduce participants to NIH grant writing and research management. The first week features presentations of general interest to new investigators, including (1) the structure of an NIH grant; (2) specific aims; (3) biosketches; (4) budgets; and (5) manuscript writing. During the second week, investigators have a choice of sessions in 3 tracks – basic science, clinical research, and community research (Table 1).

**Table 1.** Mentoring Bootcamp topics and training tracks

General Topics Week 1	Training Tracks Week 2		
	Basic Research Track	Clinical Research Track	Community Research Track
Structure of a grant NIH biosketch Budgets Manuscript writing Writing specific aims Breakout groups for basic, clinical, and community researchers	Effective lab management Communicating basic and translational science Mentor-mentee relationships Incorporating data science tools Research resources and core services	NIH clinical research funding and the peer review process Regulatory essentials Clinical protocol development and randomization Conducting clinical research Health policy basics	NIH community research funding opportunities Community-based participatory research Building academic-community partnerships Working with Native Hawaiian, Pacific Islander, and Filipino communities

The **Specific Aims Workshop** is a multi-session program offered in October, after the beginning of the academic year. During the first session, investigators are introduced to the anatomy of an NIH specific aims page and asked to prepare or revise their specific aims page within the week. Individuals attending the initial session are placed into facilitated peer groups with a maximum of 4 investigators per group. A program assistant helps each peer-group facilitator organize the timing of the follow-up meetings. Facilitators and peers provide feedback on group members’ evolving specific aims page over 2 or 3 follow-up meetings. Attendees then use their aims to prepare proposals to NIH or other funders and/or as part of their application to GUMSHOE.

**GUMSHOE** is a grant-writing-training program created by the University of Colorado and the University of Washington, initially funded through the NIH National Research Mentoring Network [6,8]. As originally offered, GUMSHOE included a 3-day intensive in-person session on grant writing, followed by work with mentors over 6 months to develop an NIH grant [17]. With permission and involvement of its originators, PIKO adapted GUMSHOE for Hawai‘i investigators by breaking the didactic portion of the training into modules and offering it on Zoom. Offered from January to May each year, the Hawai‘i version includes six 3-hour sessions and a mock review. The training focuses on writing NIH grants, reflecting the priority of the funder.

The GUMSHOE application requires participants to submit a specific aims page, identify an NIH mechanism, and specify a content mentor with whom they will work. Most applicants are accepted, and those who are not are asked to strengthen their application and apply next year. Selected participants, limited to 12 per year, attend sessions that cover the structure of a grant, how to connect with an NIH project officer, the significance, innovation, approach, and human subjects sections of a grant, the biosketch, budget, and the Application Submission System & Interface for Submission Tracking submission portal and process. Two lectures are provided on the approach section – one related to section components (e.g., sample, measures, analysis) and the other related to the importance of tailoring the approach to the community and culture of interest. Consistent with building ‘thinkforce’ capacity, a unique feature of GUMSHOE is training on integrating cultural and community perspectives and processes into the conceptual and theoretical approaches guiding the research and the research design itself (Table 2).

**Table 2.** GUMSHOE topics, content, and assignments

	Lecture Topic (1 hour)	Breakout Review Group (2 hours)	Homework
1/12	Introduction and ground rules, grant mechanisms, NOFO, PO, SA, grant structure	Review SA, grant mechanisms, NOFO and PO contact	Refine and revise SA based on feedback; review with mentors
1/26	Significance and Innovation section	Review revised SA and information from PO	Refine SA; draft Significance section; review with mentors
2/9	Approach section including investigative team, preliminary studies, and methods. Mentoring plan for K awards	Review Significance and Innovation section	Refine previous sections; draft Innovation section; review with mentors
2/23	Approach section including cultural adaptation	Review Approach section	Refine previous sections; draft Approach section; review with mentors
3/15	Biosketch, budget, IRB, support letters	Re-review Approach section and mentoring plan for K awards	Refine previous sections; draft biosketch, budget, IRB, support letters; review with mentors
4/5	Other forms and documents required in the Application Submission System & Interface for Submission Training. prepare for mock review	Review entire Research Plan, budget, letters, biosketches, IRB	Refine previous sections and start preparing forms required for submission
5/1 – 5/12	May 1 - Proposals due	May 10 - Mock review by NIH funded researchers	Revise grant based on mock review feedback and submit

**Legend:** NOFO = Notice of Funding Opportunity; PO = Program Officer; SA = Specific Aims; IRB = Institutional Review Board.

Each 60-minute lecture is followed by a 2-hour breakout group of 4 investigators and 2 facilitators, during which the evolving application is reviewed and critiqued. For example, the second lecture focuses on writing the significance and innovation sections. Participants then draft these sections with guidance from their content mentors and upload them for review by their breakout group members. At the next GUMSHOE session, members of the breakout group provide each other feedback on these sections. An optional session on building a training plan is offered for investigators writing K-series grants. Full grant applications are due in mid-April and sent to external reviewers in preparation for the mock review in early May. Investigators, facilitators, and reviewers participate in a mock study session via video conferencing facilitated by a member of the PD Core. Investigators are encouraged to improve their grants based on reviewer comments and may request that their application be re-reviewed by the same external reviewer before submission to NIH. Mock reviewers from other institutions are compensated for their time and effort.

**Annual meetings** are held with investigators between July and September of each year. Meetings are guided by a template that solicits information on the investigator’s progress to date in conducting research, disseminating findings, submitting grants, and completing activities outlined in their IDPs. Annual meetings offer an opportunity to discuss barriers to progress, identify resources that might help advance investigators’ career development, celebrate their successes, and suggest ways to improve the scaffolded training series. Additionally, personalized mentoring by members of the PD Core is provided whenever requested.

## Methods

Our evaluation included qualitative and quantitative approaches to assess and improve the process and outcomes of our training series and measure progress toward the PD Core’s overall objectives. Evaluation data were collected and managed in REDCap, with reports prepared by PIKO’s Tracking and Evaluation Core.

For each session attended, Mentoring Bootcamp participants evaluated the topic’s usefulness and the speaker’s preparedness, using a 3-point scale (3 = very; 2 = somewhat; 1 = not). For the Specific Aims Workshop, participants assessed the facilitator’s ability to lead the group and provide feedback, the usefulness of having peers review their aims pages, and the motivational value of the workshop on a 5-point Likert scale (from 5 = strongly agree to 1 = strongly disagree). Additional items ask about the usefulness of the workshop (yes/no), if participants would recommend the workshop to others (yes/no), and for suggestions on how the program could be improved.

To evaluate GUMSHOE, at the completion of the program, participants rated their level of confidence pre- and post-training with respect to 21 elements of grant writing on a 10-point scale (from 10 = total confidence to 1 = no confidence) [18]. These include refining a problem, selecting variables and data collection methods appropriate to the population, and identifying an appropriate funding mechanism. They also complete a survey asking their level of agreement with statements about the accessibility and helpfulness of facilitators, content mentors, and the different aspects of the GUMSHOE program on a 5-point scale (from 5 = strongly agree to 1 = strongly disagree). Lastly, GUMSHOE participants are asked for ways to improve the program.

We tracked longer-term outcomes in the annual mentoring meetings. For investigators completing the Specific Aims Workshop, we asked how they used their aims pages, e.g., to apply for funding or to apply to GUMSHOE. For those completing GUMSHOE, we asked about grant submission, scoring, and funding. Individuals who dropped out of the Specific Aims Workshop or GUMSHOE were interviewed to identify reasons and barriers to completion.

## Results

### Attendance

Between 2022 and 2024, 202 Hawai‘i-based investigators attended Mentoring Bootcamp (48 in 2022, 69 in 2023, and 75 in 2024), 62 unique investigators completed the Specific Aims Workshop (14 in 2022, 21 in 2023, and 27 in 2024), and 30 unique investigators completed GUMSHOE (10 per year). Investigators were allowed to repeat any training, and 14 investigators enrolled in the Specific Aims Workshop more than once, while three repeated GUMSHOE. Bootcamp was open to anyone interested in CTR. Thus, in addition to faculty, about 20% of Bootcamp attendees were students, and 13% were community researchers. In comparison, most attendees in the Specific Aims Workshop and GUMSHOE were postdoctoral fellows, assistant professors, or associate professors (∼90%) (Table 3). Participants were distributed across the basic, behavioral/community, and clinical research areas (Table 3).

**Table 3.** Rank and research area of Hawai‘i investigators in the three training programs 2022–2024

Variables	Bootcamp (N = 202)	Specific Aims Workshop (N = 62)	GUMSHOE (N = 30)
Academic Rank Student Postdoctoral Fellow Assistant Professor Associate Professor Full Professor Clinical Faculty Community Researcher	40 (20%) 40 (20%) 51 (25%) 24 (12%) 4 (2%) 17 (8%) 26 (13%)	0 10 (16%) 26 (42%) 13 (21%) 2 (3%) 7 (12%) 4 (6%)	0 6 (20%) 16 (53%) 5 (17%) 2 (7%) 1 (3%) 0
Research Area Behavioral/Community Basic Clinical	71 (35%) 73 (36%) 58 (29%)	25 (40%) 21 (34%) 16 (26%)	11 (36%) 13 (43%) 6 (20%)

### Evaluation findings

Participants in Mentoring Bootcamp could pick and choose the sessions they wished to attend. About 90% of participants attended at least one general session offered during the first week, and about 50% attended at least one specialized session during the second week. Only 33% completed the evaluation. Findings from the 2024 offering were 2.88 (range 2.7–3.0) for session usefulness and 2.93 (range 2.7–3.0) for speaker preparedness, suggesting that the sessions were well received.

Of the 62 unique investigators completing the **Specific Aims Workshop,** about 60% provided evaluation data. Findings were consistent over the 3 years. In 2024, the mean scores for the facilitators” ability to lead the group and provide feedback, for the usefulness of having peers review their aims pages, and for the motivational value of the workshop were 4.75 out of 5. Ninety percent of participants reported that the 3 or 4-session workshop helped to improve their specific aims page; 97% would attend this workshop again or recommend it to others. From annual meetings, we learned that 21 (33%) used their specific aims page in their grant application for a University of Hawai’i internally-sponsored pilot award, 4 (6%) used their aims page to apply to a private or federal funding mechanism (e.g., NIH, the US Department of Agriculture, the Health Resources and Services Administration, and Hawai‘i Community Foundation), and 16 (30%) used their aims page in their GUMSHOE applications.

All GUMSHOE participants completed the evaluation, and findings were consistently good across years. For example, in the 2024 evaluation, significant gains were observed between pre- and post-test scores in confidence performing 21 grant writing skills (Table 4). Perceptions of the availability and helpfulness of the breakout group facilitators and content mentors were positive, with mean facilitator scores ranging from 5.55 to 5.73 and mean mentor scores from 4.64 to 5.64 out of 6. Greater than 90% of participants were satisfied with the frequency and length of sessions (biweekly 1-hour lectures), the number of mentees per peer review group (4 members), and the length of breakout discussion groups (2 hours). Some participants suggested that GUMSHOE be spread out over a longer period and be designed to accommodate faculty with different levels of grant writing experience and discipline-specific needs. Of the 30 investigators who completed GUMSHOE between 2022 and 2024, 25 (83%) submitted proposals – 18 to NIH (7 funded to date), four for pilot funding from Hawai‘i-based IDeA programs (all funded), one to the National Science Foundation (funded), two to the Robert Wood Johnson Foundation (both funded), and one to the American Cancer Society (funded).

**Table 4.** GUMSHOE pre and post training survey results for 2023 and 2024 (N = 20)

How confident are you that you can successfully perform these tasks today?	PRE		POST		P*
	Mean	SD	Mean	SD	
Refine a problem so it can be investigated	4.64	1.75	7.82	1.08	0.0001
Place your study in the context of existing research and justify how it contributes to important questions in the area	4.18	1.17	7.0	1.73	0.0003
Develop a logical rationale for a particular research idea	4.73	1.79	7.64	1.5	0.001
Articulate a clear purpose for the research	5.64	1.69	7.91	1.22	0.002
Conduct research with underserved populations	4.91	1.76	7.55	1.44	0.002
State the purpose, strengths, and limitations of each study design	4.91	1.04	7.09	1.64	0.002
Relate specific questions of interests to underlying theory	5.09	1.45	7.36	1.43	0.005
Determine the universe, population, and appropriate sample for a given study	5.27	0.79	6.55	1.86	0.01
Carry out methods for conducting research with underserved populations	5.18	1.4	6.73	1.42	0.01
Organize your proposed research ideas in writing	5.45	1.92	7.45	1.63	0.02
Select a suitable topic area for study	5.55	1.51	6.91	1.67	0.03
Determine an adequate number of subjects for your research project	4.63	2.29	6.91	2.07	0.0004
Write a competitive grant application	4.63	2.111	6.72	2.14	0.002
Chose an appropriate research design that will answer a set of research questions and/or test a set of hypotheses	6.81	1.25	6.72	2.15	0.004
Speak with a person at the funding agency regarding your project or project ideas	5.18	2.04	6.54	2.25	0.01
Select methods of data collection appropriate to the study population and variable(s) of interest	5.9	0.87	7.40	1.34	0.01
Describe a major funding agency’s (e.g., NIH, foundation) proposal review and award process	4.27	1.42	7.18	2.13	0.01
Determine how each variable will be measured	6.36	1.63	5.90	0.87	0.02
Design the best data analysis strategy for your study	7.09	1.14	5.45	1.12	0.03
Convince grant reviewers your proposed study is worth funding	4.27	2.05	6.90	2.16	0.03
Identify appropriate funding sources (local, state, national) to support a study	5.81	1.07	7.09	1.13	0.03

**Legend:** P* was tabulated using the paired sample *t*-test. NIH = National Institutes of Health.

Responses to open-ended questions were positive: ‘Thank you for this excellent training opportunity,’ and ‘I was able to expand my knowledge, skills, and networks.’ Suggestions for improvement included ‘offer more opportunity for interaction in Bootcamp,’ ‘allow more time to complete the writing task in the Specific Aims Workshop,’ and ‘help us have better engagement with our content mentors.’

Six individuals enrolled in but did not complete the Specific Aims Workshop, while 3 individuals dropped out of GUMSHOE. Three of the investigators who did not complete the Specific Aims Workshop were practicing clinicians, and faculty cited heavy teaching loads as a reason for their difficulty in finding time to work on their aims and attend the review sessions. The three GUMSHOE drop-outs cited conflicting work and family-care duties.

## Discussion

The PIKO PD Core successfully developed and offered a training series using the scaffolded approach to grant writing. The training series offered dynamic support to investigators as they prepared an NIH grant. The scaffolded structure was a key characteristic of the content, resources, tasks, and mentors’ support to optimize mastery of complex grant writing skills. Macro-scaffolding was evident in the overall structure of the series, from introductory general knowledge provided through the Mentoring Bootcamp, to more refined and comprehensive information coupled with practice and feedback in the Specific Aims Workshop and GUMSHOE. Meso-scaffolding informed the steps and sequencing of activities and tasks within each training series, providing detailed instruction on each section of an NIH proposal and then asking participants to apply this information to their own work. Micro-scaffolding guided interactions between participants with small group facilitators, content mentors, and peers, with support decreasing as investigators advanced their skills and confidence [5].

Mentoring Bootcamp was the introductory offering and, while useful, subsequent hands-on training programs, like the Specific Aims Workshop and GUMSHOE, were critical to eventual grant submission. Although a few individuals dropped out of the Specific Aims Workshop and GUMSHOE, our evaluation findings, including many grant submissions, align with the educational literature and the experience of the National Research Mentoring Network that active-learning strategies, including peer and formal mock reviews, are highly effective in advancing grant writing self-efficacy and grant submissions [6,8].

All three trainings focus on NIH rules and funding mechanisms since the mission of the IDeA program is to build research capacity in states with limited NIH funding [1]. As noted, however, several investigators used their skills to apply to other funding sources. As federal support for research diminishes [19], our trainings will need to help investigators learn about other sources of research support, including professional societies, private foundations, and pharmaceutical companies.

Some investigators repeated the Specific Aims Workshop and GUMSHOE. Of the 14 who repeated the Specific Aims Workshop and the 3 who repeated GUMSHOE, most continued to refine their original aims and grant applications. Limited availability of senior facilitators restricted the number of participants in these two workshops. For example, only 8 senior faculty volunteered to facilitate breakout groups for the Specific Aims Workshop, with each assigned only 3–4 mentees. For GUMSHOE, each breakout group was facilitated by 2 senior faculty members, requiring six facilitators for 12 participants. Mock reviewers who were not employees of the University of Hawai‘i received a small stipend, but institutional policies prevented compensation for University of Hawai‘i mock reviewers and peer group facilitators.

Participation in the evaluation varied by program, ranging from about 30% for Bootcamp sessions to 100% of GUMSHOE participants. For GUMSHOE, we chose a retrospective pre-test to evaluate changes in confidence regarding grant writing. Data collected immediately after each training provided positive feedback. However, gathering longer-term outcomes required us to institute a template-guided annual meeting with each investigator to understand how training skills were being applied.

The series was time-consuming for PD Core staff, faculty, consultants, and volunteer facilitators. Mentoring Bootcamp required 100 hours to organize, manage, and deliver. The Specific Aims Workshop required 40 hours to organize and manage, and each peer-group facilitator spent an average of 10 hours reviewing and commenting on participants’ evolving aims pages. GUMSHOE was the most intensive commitment, requiring 100 hours to organize and deliver; each participant received a minimum of 40 hours of assistance from PIKO PD Core facilitators. Each annual meeting was scheduled for 1 hour, and more than 50 meetings were held in 2024. Thus, this resource-intensive training series may not be well-suited for settings without external sources of funding. An alternative may be self-paced, online courses, such as the Coursera course, Grant Writing for Health Researchers, developed by the University of Colorado [20].

Although we have characterized our experience and its process and outcomes in terms of grant writing, the nature of this enterprise is about much more than simply increasing the probability of acquiring sponsored research support. Grant writing becomes a point of reference by which new and early investigators are encouraged to systematically explore their personal and professional aspirations as scientists in the making [9]. As they traverse offerings such as those offered by PIKO, participants become equipped with the skills to assess their respective institutional landscapes, to better understand and navigate paths to success, and to identify internal as well as external allies in this process. Then, too, training series like this surface the strengths and weaknesses of the organizational settings within which they are undertaken [11]. Of long-term importance to collaboration and sustainability, intensive scaffolded training series can promote the establishment of networks of like-minded scientists who engage in CTR. The lessons we learn through PIKO highlight aspects of local culture that are critical to promoting mentees’ success. These lessons enable us to thoughtfully advocate for meaningful institutional changes in structure and processes that promise to better support the developmental trajectories of early-stage [21] and other investigators. Our experience also underscores the challenges, opportunities, and rewards of infusing such training with an authentic commitment to community-informed CTR [22].
